# Prediction of immunocyte infiltration and prognosis in postoperative hepatitis B virus-related hepatocellular carcinoma patients using magnetic resonance imaging

**DOI:** 10.1093/gastro/goae009

**Published:** 2024-02-26

**Authors:** Chenyu Song, Mengqi Huang, Xiaoqi Zhou, Yuying Chen, Zhoulei Li, Mimi Tang, Meicheng Chen, Zhenpeng Peng, Shiting Feng

**Affiliations:** Department of Radiology, The First Affiliated Hospital, Sun Yat-sen University, Guangzhou, Guangdong, P. R. China; Department of Radiology, The First Affiliated Hospital, Sun Yat-sen University, Guangzhou, Guangdong, P. R. China; Department of Radiology, Tongji Hospital, Tongji Medical College, Huazhong University of Science and Technology, Wuhan, Hubei, P. R. China; Department of Radiology, The First Affiliated Hospital, Sun Yat-sen University, Guangzhou, Guangdong, P. R. China; Department of Radiology, The First Affiliated Hospital, Sun Yat-sen University, Guangzhou, Guangdong, P. R. China; Department of Radiology, The First Affiliated Hospital, Sun Yat-sen University, Guangzhou, Guangdong, P. R. China; Department of Radiology, The First Affiliated Hospital, Sun Yat-sen University, Guangzhou, Guangdong, P. R. China; Department of Radiology, The First Affiliated Hospital, Sun Yat-sen University, Guangzhou, Guangdong, P. R. China; Department of Radiology, The First Affiliated Hospital, Sun Yat-sen University, Guangzhou, Guangdong, P. R. China; Department of Radiology, The First Affiliated Hospital, Sun Yat-sen University, Guangzhou, Guangdong, P. R. China

**Keywords:** magnetic resonance imaging, hepatocellular carcinoma, immunocyte infiltration, prognosis

## Abstract

**Background:**

The immune microenvironment (IME) is closely associated with prognosis and therapeutic response of hepatitis B virus-related hepatocellular carcinoma (HBV-HCC). Multi-parametric magnetic resonance imaging (MRI) enables non-invasive assessment of IME and predicts prognosis in HBV-HCC. We aimed to construct an MRI prediction model of the immunocyte-infiltration subtypes and explore its prognostic significance.

**Methods:**

HBV-HCC patients at the First Affiliated Hospital of Sun Yat-sen University (Guangzhou, China) with radical surgery (between 1 October and 30 December 2021) were prospectively enrolled. Patients with pathologically proven HCC (between 1 December 2013 and 30 October 2019) were retrospectively enrolled. Pearson correlation analysis was used to examine the relationship between the immunocyte-infiltration counts and MRI parameters. An MRI prediction model of immunocyte-infiltration subtypes was constructed in prospective cohort. Kaplan–Meier survival analysis was used to analyse its prognostic significance in the retrospective cohort.

**Results:**

Twenty-four patients were prospectively enrolled to construct the MRI prediction model. Eighty-nine patients were retrospectively enrolled to determine its prognostic significance. MRI parameters (relative enhancement, ratio of the apparent diffusion coefficient value of tumoral region to peritumoral region [rADC], T1 value) correlated significantly with the immunocyte-infiltration counts (leukocytes, T help cells, PD1+Tc cells, B lymphocytes). rADC differed significantly between high and low immunocyte-infiltration groups (1.47 ± 0.36 vs 1.09 ± 0.25, *P *=* *0.009). The area under the curve of the MRI model was 0.787 (95% confidence interval 0.587–0.987). Based on the MRI model, the recurrence-free time was longer in the high immunocyte-infiltration group than in the low immunocyte-infiltration group (*P *=* *0.026).

**Conclusions:**

MRI is a non-invasive method for assessing the IME and immunocyte-infiltration subtypes, and predicting prognosis in post-operative HBV-HCC patients.

## Introduction

Liver cancer is the third-leading cause of cancer-related deaths worldwide, and hepatocellular carcinoma (HCC) accounts for ∼90% of primary liver cancers [1]. The tumor immune microenvironment (IME) plays an important role in tumorigenesis and development. It is closely related to the prognosis and therapeutic response [2]. Most HCCs occur secondary to chronic liver disease. In China, the most common cause is hepatitis B virus (HBV) infection-induced hepatitis [3]. The HBV deoxyribonucleic acid (DNA) replication level also influences the immune state of the liver and HCC development [4, 5], which is probably related to the change in the IME [3].

IME research provides new immunotherapy targets for HCC treatment. However, the complexity of IME and tumor heterogeneity may cause primary or secondary treatment resistance [6]. To reflect the comprehensive functions of the IME better, combinatorial immune parameters, rather than individual parameters, have been used to classify the immunocyte infiltration into subtypes and predict patient prognosis [7, 8]. A pretreatment comprehensive assessment of immunocyte infiltration can help select the appropriate immunotherapy strategy and improve prognosis. Current methods for IME assessment, such as immunohistochemistry and gene sequencing, require invasive post-operative tissue biopsy or surgical pathology. Over half of the HCC patients are diagnosed in their advanced stages, and they may miss the opportunity for radical surgery. Furthermore, the heterogeneity of HCC cases could mislead the results. It is critical to find a dynamic monitoring method for the non-invasive and overall assessment of HCC patients throughout the diagnostic and treatment process.

Multi-parametric magnetic resonance imaging (MRI) using the hepatobiliary-specific contrast agent gadoxetic acid has been widely used in HCC for early diagnosis, tumor staging, biological behavior assessment, and prognosis prediction [9]. It has been proven a clinically valuable method for preoperative evaluation of HCC [9, 10]. Multi-parametric MRI can provide information regarding the cell density and vascular and metabolic characteristics of HCC. Some MRI sequences also allow the quantification and analysis of fat, fibrosis, and water and iron content [10]. In our previous study, we successfully predicted microvascular invasion (MVI) using gadoxetic acid-enhanced MRI [11]. Furthermore, we established a radiomics model by applying the MRI characteristics in the hepatobiliary phase, which can predict the intratumoral immune score of HCC based on the T-cell and CD3+CD8+ cell infiltration counts [12].

This study hypothesized that multi-parametric MRI with the hepatobiliary-specific contrast agent gadoxetic acid enables the non-invasive assessment of the IME. We aimed to construct an MRI prediction model of the immunocyte-infiltration subtypes and explore their prognostic significance in hepatitis B virus-related hepatocellular carcinoma (HBV-HCC) patients. Moreover, we would analyse the impact of the HBV DNA replication level on immunocyte infiltration.

## Materials and methods

### Patients

This study conformed to the principles of the Declaration of Helsinki and it was approved by the Institutional Review Board of the First Affiliated Hospital of Sun Yat-sen University (Guangzhou, China). Written informed consent was obtained from the prospectively enrolled patients and waived for retrospectively enrolled patients (Ethical approval number: NO. [2021] 208).

Patients with a clinical diagnosis of HBV-HCC who underwent radical surgery at the hospital from 1 October to 30 December 2021 were prospectively enrolled. All patients underwent gadoxetic acid-enhanced MRI within 2 weeks before the surgery. The exclusion criteria were as follows: (i) post-operative pathological diagnosis of non-HCC; (ii) poor-quality MRI scans; and (iii) insufficient tissue samples for immunocyte-infiltration analysis.

**Figure 1.**
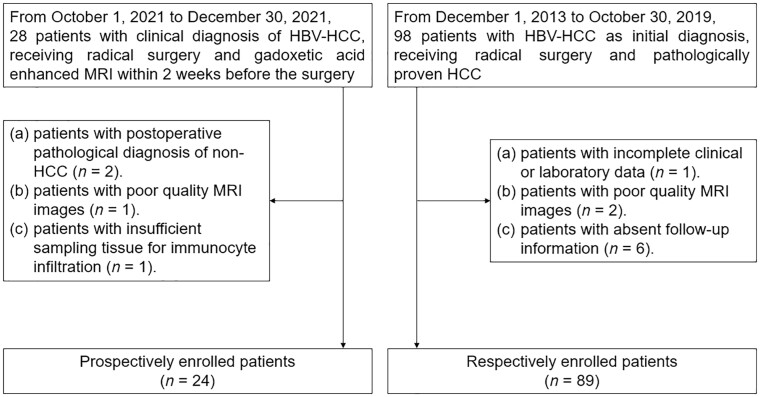
Flow diagram of the inclusion and exclusion process in the prospective (left) and retrospective (right) cohorts. HBV = hepatitis B virus, HCC = hepatocellular carcinoma, MRI = magnetic resonance imaging.

Data from patients initially diagnosed with HBV-HCC who underwent radical treatment for pathologically proven HCC at our hospital from 1 December 2013 to 30 October 2019 were retrospectively collected. All patients were followed up until 31 December 2020. The exclusion criteria for retrospectively enrolled patients were as follows: (i) incomplete clinical or laboratory data; (ii) poor-quality MRI scans; and (iii) missing follow-up data. The enrollment process is illustrated in Figure 1.

Patients who met the following two conditions were considered to have HBV-HCC: (i) clinical suspicion of HCC and (ii) history of HBV infection or indication of HBV infection on laboratory tests.

The HBV DNA replication levels within 2 weeks before radical surgery were recorded. The HBV DNA replication level was reflected by the amount of HBV DNA in the blood, which was measured quantitatively in the laboratory. In the retrospective cohort, two patients had missing HBV DNA replication level information.

The follow-up plan included serum alpha-fetoprotein (AFP) assessment and imaging examinations (ultrasound, computed tomography [CT], and MRI). These evaluations commenced 1 month after treatment and were repeated every 3–4 months during the first year, followed by subsequent assessments every 4–6 months annually [13]. Recurrence was confirmed using enhanced CT or MRI. Recurrence-free survival (RFS) was defined as the time interval between radical resection and tumor relapse [13]. The RFS and follow-up durations were recorded.

Hepatic fibrosis was classified according to the Scheuer system [14] as follows: S0, no fibrosis; S1, periportal fibrosis without septa; S2, periportal fibrosis with few septa; S3, periportal fibrosis with several septa; and S4, cirrhosis.

MVI was defined as the presence of a tumor in the portal vein, hepatic vein, or a large capsular vessel of the surrounding hepatic tissue lined by the endothelium visible only on microscopy [15]. MVI was evaluated into three types: M0 (no MVI), M1 (1–5 sites of MVI and located at ≤1 cm away from the tumor-adjacent liver tissue), and M2 (>5 MVI sites or at >1 cm away from the tumor-adjacent liver tissue).

The HCC grade was evaluated according to the Edmondson–Steiner grading system [16] as follows: I, high differentiation; II, moderate differentiation; III, low differentiation; IV, no differentiation. In the retrospective cohort, tumor grade information was missing for seven patients.

### Protocol of gadoxetic acid-enhanced MRI

Multi-parametric MRI images of the prospectively enrolled patients were acquired using a 3.0-T magnetic resonance scanner (MAGNETOM Prisma, Siemens, Germany) with an intravenous bolus injection of 0.025 mmol/kg of gadoxetic acid (Primovist, Bayer, Germany). Multi-parametric MRI images of the retrospectively enrolled patients were acquired using a 3.0-T magnetic resonance scanner (MAGNETOM Verio, Siemens) with an intravenous bolus injection of 0.025 mmol/kg of gadoxetic acid.

All patients fasted for 4 h before the examination and MRI scanning was performed in the supine position. MRI sequences of the prospective and retrospective cohort patients included the following: DIXON, T1-VIBE-fat-suppression, T1-mapping (before contrast injection), post-contrast dynamic 3D-VIBE-T1-weighted imaging (arterial phase, venous phase, and portal phase), T2WI, T2WI-fat-suppression, ep2d_diff_DKI (b = 0, 50, 400, 800 s/mm^2^), T1-mapping (20 min after contrast injection), and hepatobiliary phase T1-VIBE (20 min after contrast injection). T1-mapping consisted of a dual flip-angle 3D gradient echo sequence with a repetition time of 5.01 ms and an echo time of 2.3 ms. Quantitative T1 maps were automatically reconstructed at a post-processing workstation (Siemens).

### MRI analysis

**Figure 2.**
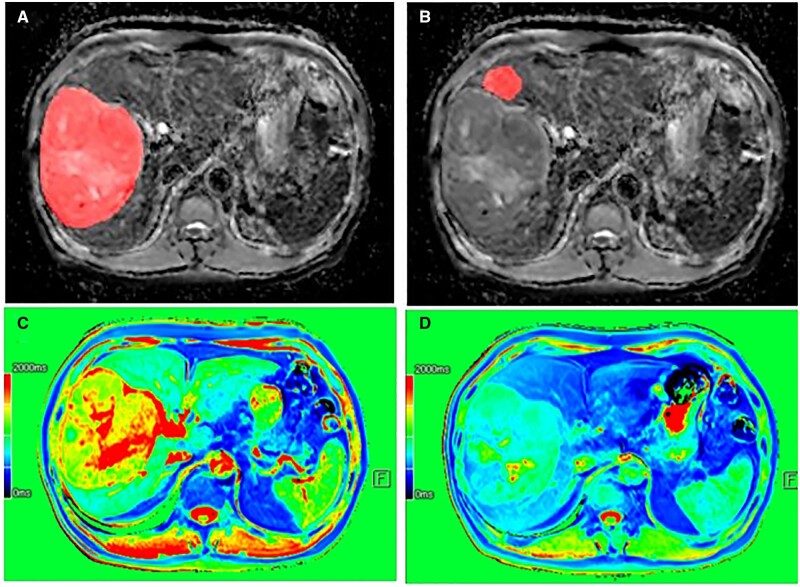
The region of interest in the tumoral and peritumoral areas. (A) Tumor delineation (marked area) on the ADC MR image; (B) peritumoral area delineation (marked area) on the ADC MR image (an area of diameter 2 cm, avoiding blood vessels and bile ducts is selected); (C) T1-mapping image before contrast injection (T1 value is 1,515.59 ms before contrast injection); (D) T1-mapping after contrast injection (T1 value is 1,156.65 ms after contrast injection). ADC = apparent diffusion coefficient, MR = magnetic resonance.

The MRI parameters were independently measured by two experienced radiologists with >5 years of work experience using an MRI post-processing workstation (Syngo.via and MR DCE v1.1.2, Siemens). The maximum plane of each tumor was used to represent the tumor region. We delineated the region of interest (ROI) on the maximum axial plane along the contour of the maximum axial plane of each tumor. The region adjacent to each tumor was used to represent the peritumoral region, avoiding the bile ducts and blood vessels. We delineated an ROI measuring 2 cm in diameter adjacent to the tumor, defined as the peritumoral region (Figure 2). The MRI parameters included T1 relaxation time before enhancement (T1_pre_), T1 relaxation time in the hepatobiliary phase (T1_pos_), signal intensity before enhancement (SI_pre_), signal intensity in the arterial phase (SI_a_), signal intensity ratio of the tumoral region to the peritumoral region in the hepatobiliary phase (SI_HBP_), and apparent diffusion coefficient (ADC) value in the tumoral and peritumoral regions (p_ADC). The relative enhancement in the arterial phase (RE), reduction rate of the T1 relaxation time (T1_ratio_), and ratio of the ADC values (rADC) were calculated as follows:

(1) $${T1}_{\text{ratio}}=\left({T1}_{\text{pre}}\;-{\;T1}_{\text{pos}}\right)/{T1}_{\text{pre}}$$

(2) $$\text{RE}={\;\text{SI}}_{a}/{\text{SI}}_{\text{pre}}$$

(3) rADC=ADC/p_ADC

### Analysis of immunocyte infiltration

The tumoral and peritumoral tissue samples were collected during surgery to analyse the immunocyte infiltration in the prospective cohort using flow cytometry. Details are provided in the Supplementary Materials. The number of infiltrating immunocytes in the tumoral and peritumoral tissues was measured using CytoFLEX flow cytometry (Beckman Coulter, USA), with a sample size of 1 × 10^5^ cells per tube. Data were obtained and analysed using CytExpert software to calculate the relative counts of the leukocytes, T lymphocytes, cytotoxic T lymphocytes, T helper cells, B lymphocytes, macrophages, PD1+T cells, PD1+CD8+T cells, PD1+Tc cells, and Treg cells. The immunocyte-infiltration ratios were calculated as follows:

(4) Leucocytes=CD45 + cell count/total cell count

(5) T lymphocytes=CD3 + cell count/CD45 + cell count

(6) B lymphocytes=CD19 + cell count/CD45 + cell count

(7) Macrophages=CD11b + CD68 + cell count/CD45 + cell count

(8) T helper cells=CD4 + cell count/CD3 + cell count

(9) Cytotoxic T lymphocytes=CD8 + cell count/CD3 + cell count

(10) PD1 + T cells=CD3 + PD1 + cell count/CD3 + cell count

(11) PD1 + Tc cells=PD1 + CD8 + cells count/CD3 + cells count

(12) Treg cells=CD3 + CD4 + CD25 + FOXP3 + cell count/CD3 + cell count

(13) PD1 + CD8 + cells=PD1 + CD8 + cell count/CD3 + CD8 + cell count

### Statistical analysis

Statistical Product and Service Solutions (version 26.0, IBM) and R software (version 4.1.2, R Foundation for Statistical Computing) were used for statistical analysis and graph drawing; *P*-values of <0.05 were considered statistically significant. Data are presented as means ± standard deviations for normal distribution, medians (interquartile ranges) for skewed distribution, and frequencies for categorical variables. For intergroup comparisons, the chi-square test, Fisher exact test, paired *t*-test, *t*-test, and rank-sum test were used. The interclass correlation coefficient (ICC) was used to determine the intra-observer consistency in the MRI quantitative parameters. Pearson or Spearman correlation analysis was used for correlation analysis. Cluster analysis was used to group patients based on their immunocyte-infiltration counts [8, 17]. The receiver-operating characteristic curve and multivariate logistic regression analysis were used to construct the MRI model for assessing immunocyte-infiltration subtypes. The retrospectively enrolled patients were grouped using the MRI model and the Kaplan–Meier analysis was used to compare patient survival.

## Results

### Clinical characteristics

The prospectively enrolled 24 HBV-HCC patients comprised 23 men and 1 woman. The retrospectively enrolled 89 HBV-HCC patients included 79 men and 10 women. Among the retrospectively enrolled 89 HBV-HCC patients, 46 experienced recurrence within 1 year after radical surgery, while 43 showed no relapse within 1 year after radical surgery.

### Immunocyte infiltration in HBV-HCC

The types and relative counts of infiltrating immunocytes in the tumoral and peritumoral tissues were analysed in the prospective cohort (Table 1). The relative leukocyte count in the tumoral tissue was significantly lower than that in the peritumoral tissue (18.39% ± 21.18% vs 25.38% ± 17.57%, *P *=* *0.026). In the tumoral tissue, T lymphocytes accounted for 70.98% ± 16.57% of leukocyte infiltration, followed by macrophages (4.80% ± 7.42%) and B lymphocytes (3.48% ± 2.69%). In the peritumoral tissue, T lymphocytes accounted for 74.74% ± 10.65% of leukocyte infiltration, followed by B lymphocytes (4.20% ± 2.31%) and macrophages (1.68% ± 1.85%). Additionally, in T-lymphocyte infiltration, the relative count of infiltrating cytotoxic T lymphocytes was significantly higher than that of T helper cells in the tumoral (36.79% ± 13.18% vs 10.46% ± 18.49%, *P *<* *0.001) and peritumoral (42.99% ± 18.68% vs 7.26% ± 5.65%, *P *<* *0.001) tissues. The relative counts of infiltrating PD1+T cells, PD1+CD8+ cells, and Treg cells were significantly higher in the tumoral tissue than in the peritumoral tissue (29.80% ± 13.41% vs 23.72% ± 9.02%, *P *=* *0.009; 46.22% ± 23.20% vs 18.79% ± 10.64%, *P *<* *0.001; and 3.29% ± 3.56% vs 2.01% ± 2.46%, *P *=* *0.010, respectively). These results revealed significant differences in the relative counts of infiltrating immunocytes between the tumoral and peritumoral tissues.

**Table 1.** Immunocyte infiltration in tumoral and peritumoral tissues in the prospective cohort of HBV-HCC patients (*n *=* *24)

Infiltrating cell type	Percentage of infiltrating immunocytes (%, mean ± SD)		*P*-value
	Tumoral tissue	Peritumoral tissue	
Leukocytes	18.39 ± 21.18	25.38 ± 17.57	0.026*
T lymphocytes	70.98 ± 16.57	74.74 ± 10.65	0.245
B lymphocytes	3.48 ± 2.69	4.20 ± 2.31	0.278
Macrophages	4.80 ± 7.42	1.68 ± 1.85	0.066
T helper cells	10.46 ± 18.49	7.26 ± 5.65	0.424
Cytotoxic T lymphocytes	36.79 ± 13.18	42.99 ± 18.68	0.166
PD1+T cells	29.80 ± 13.41	23.72 ± 9.02	0.009**
PD1+Tc cells	23.01 ± 13.46	19.71 ± 12.29	0.274
Treg cells	3.29 ± 3.56	2.01 ± 2.46	0.010*
PD1+CD8+ cells	46.22 ± 23.20	18.79 ± 10.64	<0.001***

* *P *<* *0.05;

** *P *<* *0.01;

*** *P *<* *0.001. SD = standard deviation, HBV-HCC = hepatitis B virus-related hepatocellular carcinoma.

Based on the HBV DNA replication level, we divided the prospective cohort of 24 HBV-HCC patients into the HBV DNA^+^ group (≥100 IU/mL, *n *=* *16) and the HBV DNA^–^ group (<100 IU/mL, *n *=* *8). No statistical differences were observed in sex, age, AFP level, MVI, and tumor size between these two groups (Supplementary Table 1). The degree of liver fibrosis was more severe in the HBV DNA^+^ group than in the HBV DNA^–^ group (*P *=* *0.013). When comparing the types and relative counts of infiltrating immunocytes between the HBV DNA^+^ and HBV DNA^–^ groups, only the relative count of cytotoxic T lymphocytes differed significantly between them (28.78% ± 12.09% vs 40.80% ± 12.10%, *P *=* *0.032). No significant differences were found in the relative counts of the other infiltrating immunocytes (Table 2).

**Table 2.** Comparing immunocyte infiltration in the tumoral tissue between groups stratified according to the hepatitis B virus deoxyribonucleic acid replication level in the prospective cohort

Infiltrating cell type	Percentage of infiltrating immunocytes (%, mean ± SD)		*P*-value
	HBV DNA^–^ group (*n *=* *8)	HBV DNA^+^ group (*n *=* *16)	
Leukocytes	24.60 ± 27.75	15.28 ± 17.25	0.320
T lymphocytes	78.13 ± 13.85	67.41 ± 17.05	0.138
B lymphocytes	4.18 ± 3.19	3.12 ± 2.43	0.377
Macrophages	3.15 ± 5.25	4.98 ± 7.61	0.551
T helper cells	20.02 ± 29.69	5.68 ± 6.28	0.217
Cytotoxic T lymphocytes	28.78 ± 12.09	40.80 ± 12.10	0.032*
PD1+T cells	26.21 ± 12.31	36.97 ± 13.35	0.062
PD1+Tc cells	29.82 ± 13.79	19.60 ± 12.33	0.079
Treg cells	3.04 ± 3.08	3.42 ± 3.86	0.813
PD1+CD8+ cells	47.86 ± 27.96	45.39 ± 21.41	0.812

HBV DNA^+^ = HBV DNA replication level ≥100 U/mL, HBV DNA^–^ = HBV DNA replication level <100 U/mL.

* *P *<* *0.05. SD = standard deviation, HBV = hepatitis B virus, DNA = deoxyribonucleic acid.

### Correlation analysis between MRI parameters and immunocyte infiltration

The MRI parameters were independently measured by two radiologists and showed good general consistency (ICC 0.653–0.992, *P *<* *0.05) (Supplementary Table 2).

**Figure 3.**
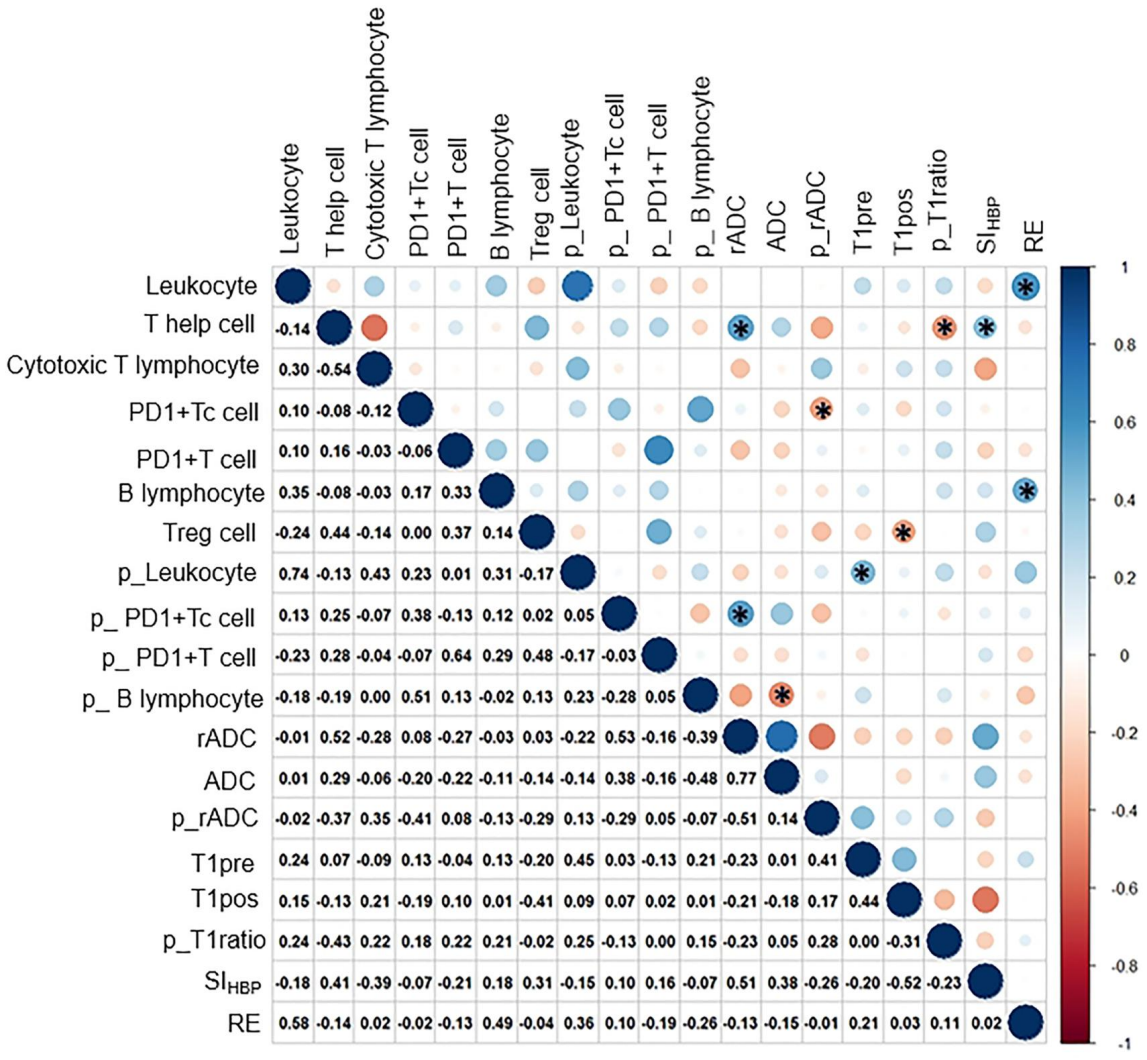
Correlation analysis between magnetic resonance imaging parameters and the relative counts of infiltrating immunocytes in the prospective cohort. The numbers provided represent the correlation coefficient of the horizontal and vertical coordinates. * indicates a significant correlation between the MRI parameters and the relative counts of infiltrating immunocytes. MRI = magnetic resonance imaging; p_Leukocyte = leukocytes in the peritumoral tissue; p_PD1+Tc cell = PD1+Tc cells in the peritumoral tissue; p_PD1+ T cell = PD1+ T cells in the peritumoral tissue, p_B lymphocyte = B lymphocytes in the peritumoral tissue.

Positive correlations were observed between the RE and the relative counts of leukocytes and B lymphocytes (*r *=* *0.58 and 0.49, respectively), between the SI_HBP_ and the relative T helper cell count (*r *=* *0.41), between the T1_pre_ and the relative leukocyte count in the peritumoral tissue (*r *=* *0.45), and between the rADC and the relative count of T helper cells and PD1+Tc cells in the peritumoral tissue (*r *=* *0.52 and 0.53, respectively) (*P *<* *0.05). Negative correlations were observed between the T1_ratio_ in the peritumoral tissue and the relative T helper cell count (*r *=* *–0.43), between the T1_pos_ and the relative Treg cell count (*r *=* *–0.41), between the p_ADC and the relative PD1+Tc cell count (*r *=* *–0.41), and between the ADC and the relative B lymphocyte count in the peritumoral tissue (*r *=* *–0.48) (*P *<* *0.05). Significant correlations were observed between the MRI parameters and the relative counts of infiltrating immunocytes in both tumoral and peritumoral tissues. The correlation analysis between the MRI parameters and the relative counts of immunocyte infiltration is shown in Figure 3.

### MRI model of immunocyte infiltration

**Figure 4.**
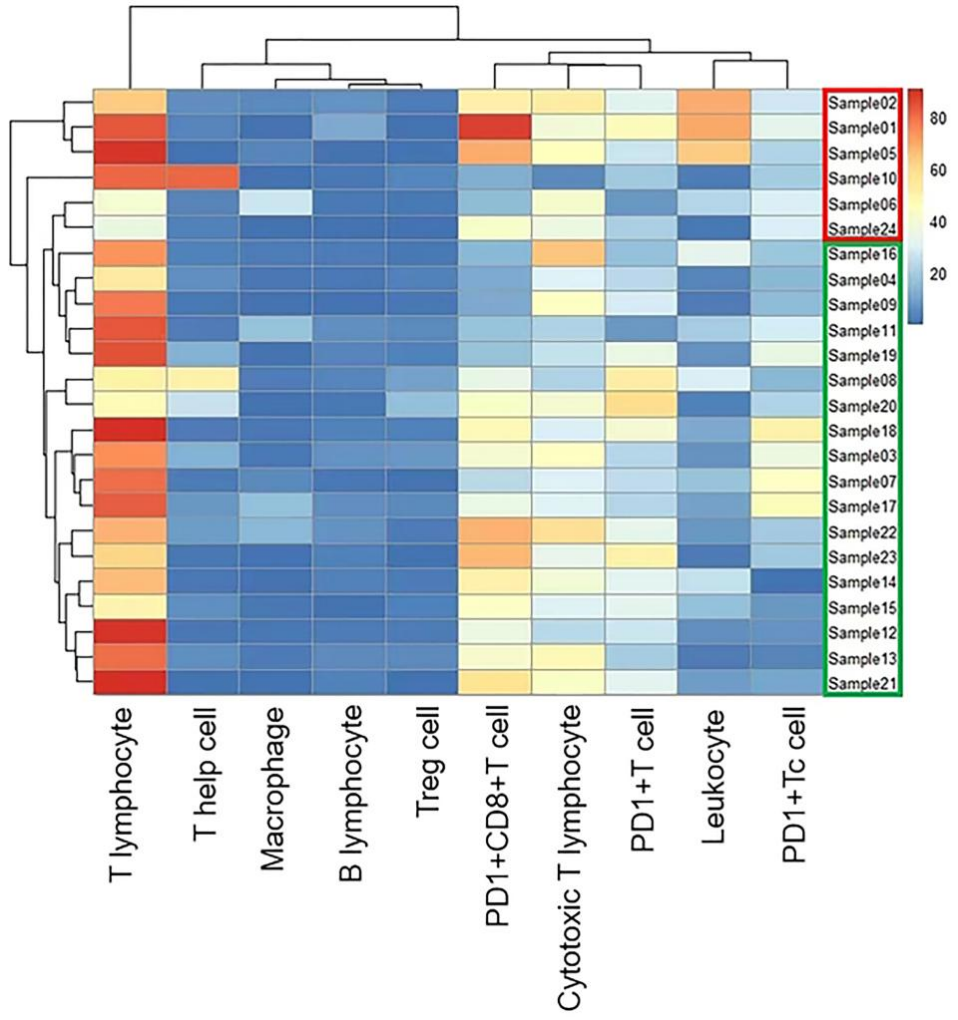
Cluster analysis of immunocyte infiltration in the prospective cohort. Based on immunocyte infiltration, 24 patients were divided into the high immunocyte-infiltration group (upper box; sample 02, 01, 05, 10, 06, and 24; *n *=* *6) and the low immunocyte-infiltration group (lower box; sample 16, 04, 09, 11, 19, 08, 20, 18, 03, 07, 17, 22, 23, 14, 15, 12, 13, and 21; *n *=* *18).

Based on the relative counts of the infiltrating immunocytes, the prospectively enrolled patients were stratified into the high and low immunocyte-infiltration groups using cluster analysis (Figure 4). Supplementary Table 3 shows the comparison of immunocyte infiltration between the high and low immunocyte-infiltration groups in the prospective cohort.

**Figure 5.**
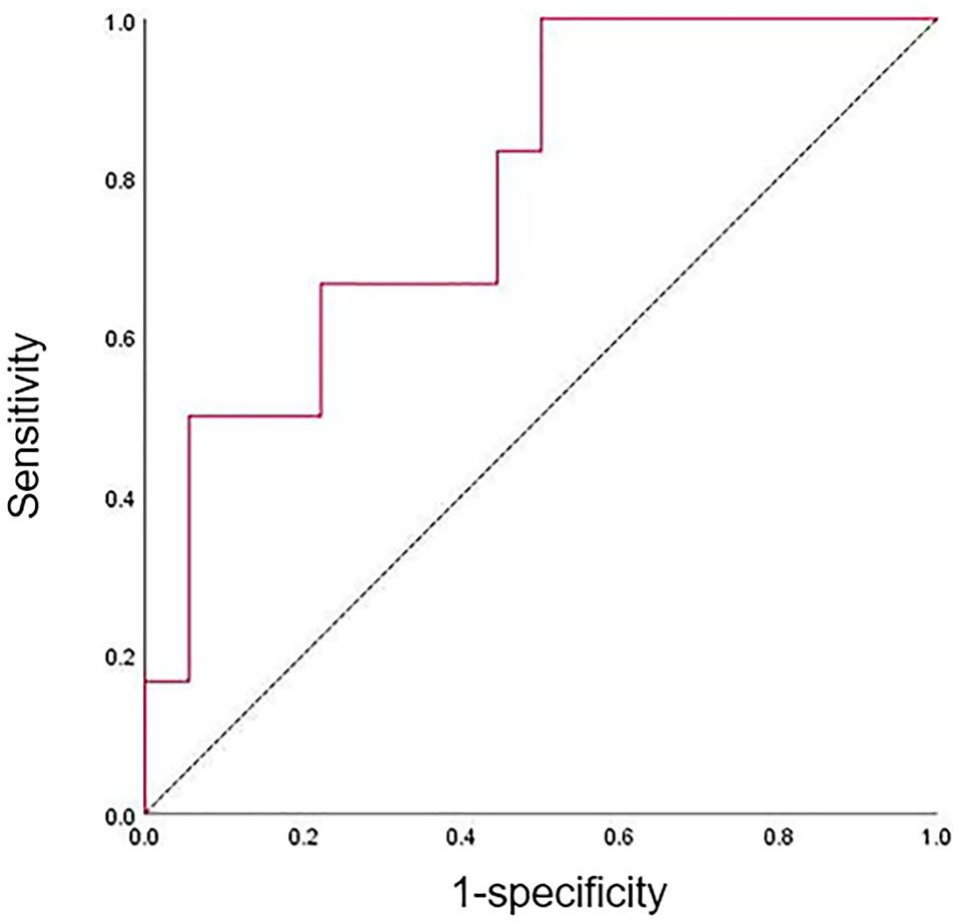
The receiver-operating characteristic curve of the magnetic resonance imaging prediction model of the immunocyte-infiltration subtypes in the prospective cohort. The area under the curve is 0.787 (95% CI 0.587–0.987). When the rADC is 1.10, the sensitivity is 83.3% and the specificity is 55.6%. CI = confidence interval, rADC = ratio of the apparent diffusion coefficient value.

The baseline clinical characteristics of the prospectively enrolled HBV-HCC patients in the high and low immunocyte-infiltration groups were compared (Table 3). Moreover, the MRI parameters of the prospective cohort in the high and low immunocyte-infiltration groups were compared (Table 4). The rADC differed significantly between the high and low immunocyte-infiltration groups (1.47 ± 0.36 vs 1.09 ± 0.25, *P *=* *0.009). To establish the MRI prediction model of immunocyte-infiltration subtypes, the rADC values of the high and low immunocyte-infiltration groups of the prospective cohort were included. The area under the curve was 0.787 (95% confidence interval 0.587–0.987) for stratifying the immunocyte-infiltration subtypes in the prospective cohort. When the rADC was 1.10, the sensitivity and specificity of the model were 83.3% and 55.6%, respectively (Figure 5).

**Table 3.** Comparison of baseline clinical characteristics between the high and low immunocyte-infiltration groups of the prospective cohort and between the retrospective and prospective cohorts

Clinical characteristic	Low immunocyte-infiltration group (*n *=* *18)	High immunocyte-infiltration group (*n *=* *6)	*P^a^*-value	Prospective cohort (*n *=* *24)	Retrospective cohort (*n *=* *89)	*P^b^*-value
Sex, *n* (%)						
Male	17 (94.44)	6 (100.00)	1.000	1 (4.17)	10 (11.24)	0.516
Female	1 (5.56)	0 (0.00)		23 (95.8)	79 (88.76)	
Age, years, mean ± SD	49.87 ± 10.58	55.33 ± 9.05	0.267	51.21 ± 10.32	52.36 ± 12.47	0.646
HBV DNA, *n* (%)						
-	5 (27.78)	3 (50.00)	0.362	8 (33.33)	48 (55.17)	0.313
+	13 (72.22)	3 (50.00)		16 (66.67)	39 (44.83)	
AFP, μg/L, median (IQR)	144.81 (1,625.96)	10.29 (39.39)	0.07	85.05 (494.70)	26.28 (504.50)	0.811
HCC grade, *n* (%)						
II	8 (44.44)	4 (66.67)	0.640	12 (50.00)	54 (65.85)	0.159
III	10 (55.56)	2 (33.33)		12 (50.00)	28 (34.15)	
MVI, *n* (%)						
0	10 (55.56)	4 (66.67)	0.496	/	/	/
1	5 (27.78)	2 (33.33)		/	/	
2	3 (16.67)	0 (0.00)		/	/	
Cirrhosis, *n* (%)						
S0	2 (11.11)	0 (0.00)	0.427	/	/	/
S1	1 (5.56)	2 (33.33)		/	/	
S2	6 (33.33)	2 (33.33)		/	/	
S3	3 (16.67)	1 (16.67)		/	/	
S4	6 (33.33)	1 (16.67)		/	/	

*P* ^a^ = *P*-value indicates the significance level of the comparison between the high and low immunocyte-infiltration groups.

*P* ^b^ = *P*-value indicates the significance level of the comparison between the prospective and retrospective cohorts.

HBV = hepatitis B virus, DNA = deoxyribonucleic acid, HBV DNA^+^ = HBV DNA replication level ≥100 U/mL, HBV DNA^–^ = HBV DNA replication level <100 U/mL, AFP = alpha-fetoprotein, HCC = hepatocellular carcinoma, HCC Grade II = moderate differentiation, HCC Grade III = low differentiation, MVI = microvascular invasion, S0 = no fibrosis, S1 = periportal fibrosis without septa, S2 = periportal fibrosis with few septa, S3 = periportal fibrosis with several septa, S4 = cirrhosis.

**Table 4.** Comparison of the magnetic resonance imaging parameters between the high and low immunocyte-infiltration groups of the prospective cohort

MRI parameter	High immunocyte-infiltration group (*n *=* *6)	Low immunocyte-infiltration group (*n *=* *18)	*P*-value
T1_pre_ (ms)	1,453.62 ± 197.84	1,438.78 ± 194.70	0.874
T1_pos_ (ms)	903.82 ± 109.48	876.08 ± 197.16	0.748
T1_ratio_	0.37 ± 0.06	0.39 ± 0.12	0.811
p_T1_pre_	911.25 ± 113.66	945.11 ± 97.81	0.487
p_T1_pos_	340.47 ± 114.32	350.52 ± 96.93	0.835
p_T1_ratio_	0.62 ± 0.14	0.63 ± 0.11	0.904
rADC	1.47 ± 0.36	1.09 ± 0.25	0.009**
p_ADC (×10^–6^ mm^2^/s)	874.75 ± 99.17	993.71 ± 152.22	0.089
ADC (×10^–6^ mm^2^/s)	1,263.63 ± 212.00	1,076.07 ± 245.28	0.109
SI_HBP_	0.39 ± 0.16	0.43 ± 0.16	0.693
RE	1.21 ± 0.35	0.99 ± 0.36	0.210

** *P *<* *0.01. MRI = magnetic resonance imaging, T1_pre_ = T1 relaxation time before enhancement, T1_pos_ = T1 relaxation time in the hepatobiliary phase, T1_ratio_ = reduction rate of the T1 relaxation time, rADC = ratio of the apparent diffusion coefficient value, p_ADC = apparent diffusion coefficient value of the peritumoral region, ADC = apparent diffusion coefficient, SI_HBP_ = signal intensity ratio of the tumoral region to the peritumoral region in the hepatobiliary phase, RE = relative enhancement in the arterial phase.

**Figure 6.**
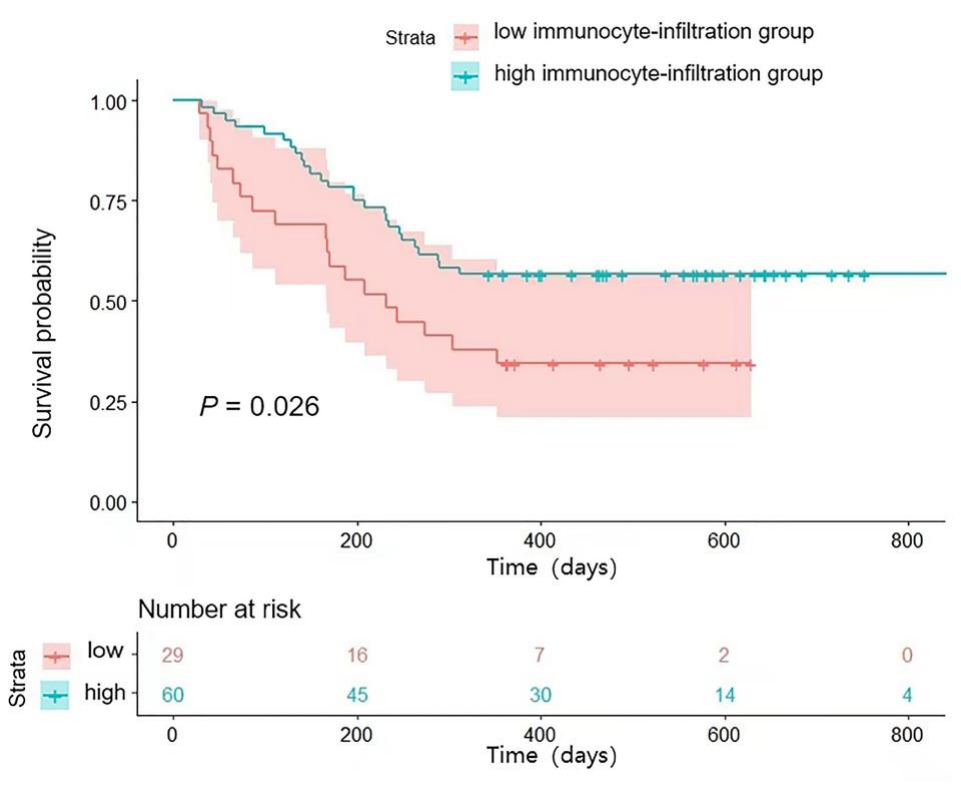
Retrospectively enrolled patients in the high immunocyte-infiltration group show significantly better recurrence-free survival than those in the low immunocyte-infiltration group assessed using the magnetic resonance imaging model (*P *=* *0.026).

The baseline clinical characteristics of the prospective and retrospective cohorts were also compared (Table 3). The retrospectively enrolled HBV-HCC patients were grouped into the high immunocyte-infiltration group (rADC ≥ 1.10, *n *=* *60) and the low immunocyte-infiltration group (rADC < 1.10, *n *=* *29), using the established MRI prediction model. Survival analysis revealed that the retrospectively enrolled HBV-HCC patients in the high immunocyte-infiltration group had significantly better RFS than those in the low immunocyte-infiltration group (*P *=* *0.026) (Figure 6 and Table 5).

**Table 5.** Survival of the high and low immunocyte-infiltration groups of the retrospective cohort

Group	Mean follow-up time (days, 95% CI)	Longest follow-up time (days)	1-year recurrence rate (%, 95% CI)	Recurrence-free rate (%, 95% CI)	Median survival time (days, 95% CI)
High immunocyte-infiltration group	407.72 (345.70–469.74)	895	48.33 (35.32–61.35)	51.67 (38.65–64.68)	/
Low immunocyte-infiltration group	264.34 (192.47–336.22)	629	72.41 (55.11–89.72)	27.59 (10.28–44.89)	232.00 (131.79–332.21)

CI = confidence interval.

## Discussion

This study provides an approach to evaluate the IME and suggests a way to predict the prognosis in HBV-HCC. The prognostic significance of the MRI prediction model was also validated in retrospectively enrolled HBV-HCC patients. A better RFS was observed in the high immunocyte-infiltration group than in the low immunocyte-infiltration group.

Considering the sample size and necrotic area inside the tumor, we used the immunocyte-infiltration ratio to determine the infiltration of various immunocytes. We defined the high and low immunocyte-infiltration groups according to the relative count of the CD45+ cells (leukocytes), which represents the total immunocyte infiltration.

The MRI parameter of rADC can predict the immunocyte-infiltration subtypes. Patients in the high immunocyte-infiltration group (rADC ≥ 1.10) had a longer RFS than those in the low immunocyte-infiltration group (rADC < 1.10). This result is consistent with those of previous studies [18, 19] that the average ADC value of HCC can predict the early recurrence of HCC after surgery. Low ADC values indicate high tumor proliferation activity and fewer necrotic areas, which are associated with poor prognosis. Although the malignancy degree and prognosis are related to various factors, immunocytes play an important role in tumor development [7, 8, 20]. In HCC, the higher the immunocyte infiltration, the better the prognosis [21]. This may be because the immune system monitors the tumor, constantly eliminating tumor antigens and reducing tumor clonal heterogeneity [22]. Therefore, patients in the high immunocyte-infiltration group in this study had significantly higher leukocyte infiltration than those in the low immunocyte-infiltration group, and the infiltrating leukocytes were dominated by T lymphocytes, which may indicate a good prognosis.

The gadoxetic acid uptake is a quantitative indicator of hepatocyte function and is related to the severity of liver fibrosis and cirrhosis. SI_HBP_ is a parameter associated with the expression of organic anion transporters in the tumoral and peritumoral tissues. The upregulation of intratumoral organic anion transporters correlates positively with signal increase, indicating well-differentiated HCC [23]. T helper cells play a central role in the adaptive immune system by facilitating the activity of other immunocytes, indicating an active immune state with an increased T helper cell count. In this study, we observed a positive correlation between the relative T helper cell count and the SI_HBP_. Well-differentiated HCC with an active immune state could be the explanation. In this study, the RE in the tumor region correlated positively with the relative infiltration counts of leukocytes and B lymphocytes. The RE in the tumoral region is related to the arterial blood supply, oxygen supply, and tumor angiogenesis, which are closely associated with the activity of leukocyte and B lymphocyte infiltration [24, 25]. A study has shown that immunocyte infiltration in tumors and their functions are related to tumor vascularization, which is closely associated with the prognosis of patients undergoing immunotherapy [26]. In this study, a negative correlation was found between the T1_pos_ and Treg cell infiltration in the tumor. Intratumoral hemorrhage and excessive interstitial components cause contrast retention. Increased paramagnetism is the most common cause of the reduction in the T1_pos_ in HCC. Intratumoral hemorrhage is associated with vasodilation, proliferation of thin-walled vessels, and tumor necrosis [27], promoting tumor growth with increased intratumoral blood vessels and inflammatory cell infiltration [28]. The interstitial component is an important member of the IME that promotes tumor progression and metastasis [29] and is associated with immunosuppression and immune escape [30]. Therefore, a reduction in the T1_pos_ may indicate an increase in the number of immunosuppressive Treg cells. We found a positive correlation between the rADC and the relative count of T helper cells. In this study, patients with rADC ≥ 1.10 belonged to the high immunocyte-infiltration group and had a longer RFS than those in the low immunocyte-infiltration group, which is consistent with the influence of increased T helper cells mentioned above. Additionally, a positive correlation was observed between the rADC and the relative count of p_PD1+Tc cells. According to the previous study [18], as the mitotic activity and nucleus/cytoplasm ratio increase, the free diffusion of water molecules in the intracellular space decreases, which leads to a reduced ADC value. ADC values were inversely correlated with restricted water diffusion [31]. An increase in the p_PD1+Tc cells indicates increased immunosuppression in the peritumoral tissue. The increase in immunosuppressive cells is related to the decrease in the free diffusion restriction of water molecules. As shown in Equation (3), rADC = ADC/p_ADC, where rADC increases as p_ADC decreases. The increase in the PD1+Tc cells indicates increased immunosuppression in the tumoral tissue, which is probably related to the increased invasive behavior of the tumor. As the tumor invaded the peritumoral tissue, the free diffusion degree of water restriction in the peritumoral tissue decreases, and the p_ADC value decreases. This could explain the negative correlation between the p_ADC and the relative count of PD1+Tc cells.

Immunocyte infiltration in the tumoral and peritumoral tissues was separately detected in this study. The tumoral tissue showed fewer leukocytes, more PD1+CD8+ cells, and more Treg cells than the peritumoral tissue, indicating more severe suppression and depletion of the immune state. The upregulation of PD1 molecule expression in T lymphocytes promotes the transformation of activated T lymphocytes into a dysfunctional state, which inhibits the antitumor activity and proliferation of T lymphocytes [32]. Treg cells in tumors can resist tumor-specific immune responses and inhibit tumor immunity by inhibiting cytotoxic T-lymphocyte infiltration [33]. However, the immunocyte-infiltration ratio in the tumoral and peritumoral tissues did not corroborate our observation. Treg cell and PD1+T cell infiltrations were more numerous in the tumoral tissue than in the peritumoral tissue, indicating more severe immunosuppression in the former than in the latter. The infiltration of the cytotoxic T lymphocytes was lower in the tumoral tissue than in the peritumoral tissues; however, the difference was not significant. Based on gene analysis, Rohr-Udilova *et al.* [34] showed that the proportions of inactivated T helper cells and macrophages play a major role in tumors. Inactivated T helper cells have the potential to differentiate into cells with different functions. Macrophages have different subtypes and play a dual role in promoting and inhibiting the tumor-killing process. The different relative counts of immunocytes among different studies could be related to various factors, such as sampling bias, variant immunocyte analysis methods, differences in HCC clustering, or the inconsistent background of chronic liver diseases.

All the patients included in this study had concomitant HBV infection. The cytotoxic T-lymphocyte infiltration was greater and liver fibrosis or cirrhosis was more severe in the HBV DNA^+^ group than in the HBV DNA^–^ group. HBV DNA levels are an important risk factor for post-operative recurrence, including early and late recurrences [35]. Lim *et al.* [36] demonstrated that there were more regulatory T lymphocytes and cytotoxic T lymphocytes, greater expression of PD1, and more T lymphocytes in a state of inhibition and depletion in HBV-HCC than in non-viral HCC. In this study, we analysed the differences in the immunocyte infiltration between the HBV DNA^+^ and HBV DNA^–^ groups. There were more cytotoxic T lymphocytes in the HBV DNA^+^ patients than in the HBV DNA^–^ patients, which could be related to the stronger immunogenicity of tumors in patients with active viral replication than in those without it. Another study [37] has shown that the proportion of PD1+CD8+ cells was high in the HBV-HCC patients. HBV infection, which is in an active HBV DNA replication state, affects the IME of HCC patients and may affect the efficacy of immunotherapy.

This study identified a correlation between the MRI parameters and relative counts of infiltrating immunocytes. We also established an MRI prediction model for the immunocyte-infiltration subtypes and validated its effectiveness in predicting prognosis. However, this study has some limitations that warrant further investigation. First, sampling bias due to tumor heterogeneity may have affected the results of flow cytometry analysis. Total tumor analysis is required to improve the accuracy of the MRI model. Second, this was a single-center study with a small sample size. Hence, multicenter studies with larger sample sizes are warranted for further analysis of multi-parametric MRI efficacy in evaluating immunocyte infiltration in HBV-HCC.

## Conclusions

MRI is an effective non-invasive method for assessing the immunocyte-infiltration subtypes and predicting prognosis. This study established an MRI predictive model for evaluating immunocyte infiltration in HBV-HCC patients, which could potentially assist in selecting the appropriate immunotherapy strategy for HBV-HCC patients and enable non-invasive monitoring of the treatment process.

## Supplementary Material

goae009_Supplementary_Data
