# Supplementary material for: Prediction of immunocyte infiltration and prognosis in postoperative hepatitis B virus-related hepatocellular carcinoma patients using magnetic resonance imaging
Source: Gastroenterol Rep (Oxf). 2024 Feb 26;12:goae009. doi: 10.1093/gastro/goae009 (PMC10898339; doi:10.1093/gastro/goae009)
Supplement: goae009_Supplementary_Data [file goae009_supplementary_data.docx]

**Analysis of immunocyte infiltration**

Human tissue samples were collected within 30 min *in vitro* and put into centrifuge tubes containing 10 mL of RPMI 1640 medium in a refrigerator at 4°C for storage immediately. Then, samples were prepared for follow-up immunocyte number analysis. The 1.0 cm × 0.5 cm × 0.3 cm tumoral sample and 1.0 cm × 0.5 cm × 0.3 cm peritumoral sample, avoiding necrotic area, were taken from all patients. The peritumoral sample was chosen in the direction of the incision margin within 2 cm away from the tumor. Flow cytometry experiment began within 1 hour after sampling. Sampling tissue was cut into pieces and digested with prepared digestive enzyme, which consists of type I (40507ES60, Yeasen, Shanghai, China) and type IV collagenase (40510ES60, Yeasen), type II dispase enzyme (40104ES80, Yeasen) and DNA digestive enzyme (D8070, Solarbio, Beijing, China), in 37℃ water-bath for 30 min. 70 μm cell mesh was used to filter the cell suspension. The cell precipitation was then collected for erythrocyte lysis (C3702, Beyotime, Shanghai, China). After centrifugation, was the cell precipitation suspend in 6 mL phosphate buffered saline (PBS, G0002, Servicebio, Wuhan, China) solution and formed a single cell suspension. This 6 mL cell suspension was evenly divided into three tubes and placed in the new flow tubes, labeled X-1, X-2, X-3. Tube X-1 was used to tag and count CD45+ cells, CD3+ cells, CD19+ cells, CD11b+CD68+/macrophages, CD3+CD4+ cells and CD3+CD8+ cells. Tube X-2 was used to tag and count CD3+CD4+CD25+Foxp3+ cells. Tube X-3 was used as the blank control to avoid false positive results. Tube X-1 and tube X-2 were centrifugalized in 4℃ with 400 g for 5 min. The cell precipitation was collected and the cell surface antibodies were added. Among them, 0.5 μL CD45 (304036, Biolegend, USA), 0.5 μL CD19 (11-0199-42, eBioscience, USA), 0.5 μL CD3 (35-0039-T100, Tonbo Biosciences, USA), 0.5 μL CD4 (65-0049-T100, Tonbo Biosciences), 0.5 μL CD8 (565289, BD Biosciences, USA), 0.5 μL CD279/PD-1 (329920, Biolegend) and 0.5 μL CD14 (325618, Biolegend) were added into tube X-1. 0.5 μL CD3, 0.5 μL CD4 and 0.5 μL CD25 (20-0259-T100, Tonbo Biosciences) were added into tube X-2. After thorough mixing, tube X-1 and tube X-2 were incubated at room temperature for 20 min, protected from light. 2 mL PBS was used to wash out the unconjugated antibodies for twice. The cell precipitation was collected and 0.8 mL immunostaining permeabilization buffer with Triton X-100 (P0096, Beyotime) was added into tube X-1 and tube X-2, respectively for 40 min, protected from light, washed using 2 mL PBS after incubation. The cell precipitation was collected and the intracellular antibodies were added. Tube X-1 was added 0.5 μL CD68 (333808, Biolegend). Tube X-2 was added 0.5 μL Foxp3 (12-4777-42, eBioscience). After thorough mixing, tube X-1 and tube X-2 were incubated at room temperature for 40 min, away from light. Then, they were wash twice with 2 mL PBS. The cell precipitation was collected and suspend in 0.5 mL PBS for flow cytometry.

**Supplementary Table 1.** The comparison of clinical characteristics between HBV DNA^+^ and HBV DNA^-^ groups in prospective cohort

| Clinical characteristic | HBV DNA^+^ group  (*n* = 16) | HBV DNA^-^ group  (*n* = 8) | *P* |
| --- | --- | --- | --- |
| Sex (male/female) | 15/1 | 8/0 | 1.000 |
| Age, years, mean ± SD | 50.31 ± 10.11 | 53.00 ± 11.19 | 0.559 |
| AFP, μg/L, median (IQR) | 116.02 (6.95–499.51) | 20.20 (4.10–1081.46) | 0.358 |
| MVI (+/-) | 8/8 | 2/6 | 0.388 |
| Tumor size, mm, mean ± SD | 69.56 ± 19.62 | 61.38 ± 41.81 | 0.612 |
| Liver fibrosis |  |  | 0.013 |
| S0 | 0 | 2 |  |
| S1 | 1 | 2 |  |
| S2 | 6 | 2 |  |
| S3 | 2 | 2 |  |
| S4 | 7 | 0 |  |

HBV DNA^+^: hepatitis B virus deoxyribonucleic acid replication active; HBV DNA^-^: hepatitis B virus deoxyribonucleic acid replication inactive; AFP: The serum alpha fetoprotein; MVI: microvascular invasion.

**Supplementary Table 2.** Consistency test of MRI parameters between observers

| Parameter | Observer 1 | Observer 2 | ICC | 95% CI | *P* |
| --- | --- | --- | --- | --- | --- |
| T1_pre_ (ms) | 1442.49 ± 191.23 | 1445.41 ± 177.67 | 0.975 | 0.942–0.989 | <0.001 |
| T1_pos_ (ms) | 883.01 ± 177.45 | 900.58 ± 183.68 | 0.940 | 0.862–0.974 | <0.001 |
| T1_ratio_ | 0.38 ± 0.11 | 0.37 ± 0.11 | 0.911 | 0.794–0.962 | <0.001 |
| pT1_pre_ (ms) | 936.64 ± 100.51 | 901.97 ± 80.97 | 0.653 | 0.198–0.850 | 0.007 |
| pT1_pos_ (ms) | 348.00 ± 99.02 | 314.84 ± 94.49 | 0.790 | 0.514–0.909 | <0.001 |
| pT1_ratio_ | 0.63 ± 0.11 | 0.65 ± 0.12 | 0.897 | 0.763–0.956 | <0.001 |
| rADC | 1.19 ± 0.32 | 1.19 ± 0.34 | 0.946 | 0.876–0.977 | <0.001 |
| ADC (10^-6^ mm^2^/s) | 1122.96 ± 247.23 | 1118.06 ± 148.43 | 0.992 | 0.981–0.996 | <0.001 |
| p_ADC (10^-6^ mm^2^/s) | 963.97 ± 148.44 | 972.26 ± 185.90 | 0.837 | 0.622–0.929 | <0.001 |
| RE | 1.05 ± 0.37 | 2.11 ± 0.47 | 0.706 | 0.320–0.873 | 0.002 |
| SI_HBP_ | 0.42 ± 0.16 | 0.44 ± 0.18 | 0.922 | 0.820–0.966 | <0.001 |

ICC: Interclass correlation coefficient; CI: Confidence interval;T1_pre_: T1 relaxion time before enhancement; T1_pos_: T1 relaxion time in hepatobiliary phase; T1_ratio_: the reduction rate of the T1 relaxion time; pT1_pre_: T1 relaxion time before enhancement in peritumoral region; pT1_pos_: T1 relaxion time in hepatobiliary phase in peritumoral region; pT1_ratio_: the reduction rate of the T1 relaxion time in peritumoral region; rADC: Rate of the apparent diffusion coefficient value; ADC: Apparent diffusion coefficient; p_ADC: Apparent diffusion coefficient value of the peritumoral region; RE: Relative enhancement in arterial phase; SI_HBP_: Signal intensity rate of the tumoral to peritumoral region in hepatobiliary phase.

**Supplementary Table 3.** The comparison of immunocyte infiltration between high and low immunocyte-infiltration groups in prospective cohort

| Infiltrating cell type | Percentage of infiltrating immunocyte  (%, mean ± SD) | | *P-*value |
| --- | --- | --- | --- |
|  | High immunocyte-infiltration group  (*n* = 6) | Low immunocyte-infiltration group  (*n* = 18) |  |
| Leukocytes | 38.30 ± 33.51 | 11.75 ± 9.49 | 0.005 |
| T lymphocytes | 65.64 ± 22.84 | 72.76 ± 14.30 | 0.374 |
| B lymphocytes | 4.19 ± 4.15 | 3.56 ± 2.26 | 0.639 |
| Macrophages | 2.52 (0.49–10.59) | 1.53 (0.49–3.19) | 0.689 |
| T help cells | 3.74 (2.56–24.71) | 4.28 (1.61–9.57) | 0.894 |
| Cytotoxic T lymphocytes | 36.90 ± 16.72 | 36.76 ± 12.36 | 0.985 |
| PD1+T cells | 25.27 ± 13.37 | 31.31 ± 13.46 | 0.351 |
| PD1+Tc cells | 26.79 ± 5.39 | 21.75 ± 15.17 | 0.439 |
| Treg cells | 1.27 ± 1.45 | 3.97 ± 3.82 | 0.109 |
| PD1+CD8+ T cells | 46.27 ± 29.65 | 36.77 ± 18.27 | 0.356 |
